# From yield to nutrition: re-imagining crop nutritional resilience for global nutrition security under climate change

**DOI:** 10.3389/fnut.2026.1987519

**Published:** 2026-10-14

**Authors:** D. M. S. B. Dissanayaka

**Affiliations:** Department of Crop Science, Faculty of Agriculture, University of Peradeniya, Peradeniya, Sri Lanka

**Keywords:** biofortification, climate change, food systems, hidden hunger, micronutrients, nutrient density

## Abstract

Climate change is increasingly challenging crop-based food and nutrition security not only through reductions in crop productivity but also by altering the nutritional composition, bioavailability, and functionality properties of food. Rising temperature, drought, excessive rainfall and flooding, elevated atmospheric CO_2_, and salinity can modify the accumulation, partitioning, and availability of proteins, minerals, vitamins, carotenoids, phenolics, flavonoids, and other bioactive compounds in crop-derived foods. Elevated CO_2_ is frequently associated with dilution of protein, iron (Fe), zinc (Zn), and other minerals in crops while heat and water stress can further disrupt nutrient uptake, translocation, and grain filling. However, responses are not universally negative: moderate environmental stress may stimulate selected phytochemicals and antioxidants, and responses depend strongly on crop species, genotype, nutrient, stress intensity and duration, developmental stage, and environmental context. Nutrient concentration alone also does not determine nutritional value because anti-nutritional factors, food matrix characteristics, processing, digestibility, and bioavailability influence the nutrients ultimately available to consumers. This mini-review synthesizes the evidence on climate-related changes in crop nutritional quality and develops the concept of nutritional resilience around four interconnected dimensions: productivity, nutrient density, bioavailability, and dietary diversity. The framework is intended to complement, not replace, existing food security, nutrition-sensitive agriculture, and climate-resilient approaches by emphasizing whether climate adaptation can maintain nutritionally meaningful food from crop production through consumption.

## 1 Introduction

Food security is now understood more broadly than the production of sufficient quantities of food. Contemporary definitions emphasize physical, social and economic access to sufficient, safe and nutritious foods that meet dietary needs and preferences, together with the dimensions of availability, access, utilization and stability (1, 2). Crop improvement has likewise expanded beyond yield alone to include nutritional quality, biofortification, stress tolerance, resource-use efficiency, and traits linked to human health. The relevant emerging challenge is therefore not simply to introduce nutrition into an otherwise yield-centred paradigm, but to determine whether climate change could erode or alter nutritional gains already achieved while agricultural systems adapt to climatic stress. Plant-derived foods provide proteins, minerals, vitamins, dietary components, antioxidants, and diverse phytochemicals, making their nutritional integrity a fundamental component of healthy and sustainable diets (1, 2). Climate change is altering the environmental conditions under which food crops develop. Increasing atmospheric CO_2_, temperature extremes, drought, excessive rainfall and flooding, and salinity can affect plant physiology, soil nutrient dynamics, metabolism, and assimilate allocation to edible tissues (3–5). Consequently, climate change can influence not only crop yield but also the concentration and composition of nutrients and bioactive compounds in harvested products (6–9).

The nutritional consequences are particularly important where diets depend heavily on a limited number of staple crops. Elevated CO_2_ has been associated with reductions in Fe, Zn, protein, and selected B vitamins in important food crops (10–12). Such changes may aggravate existing micronutrient deficiencies and hidden hunger, particularly among populations dependent on cereals and other nutrient-sensitive staples (11, 13). Climate change is projected to substantially increase the number of children affected by micronutrient and protein deficiencies, potentially adding hundreds of millions to the burden by 2050 across different age groups, including school-aged children and adolescents (14). This projected increase is particularly concerning given the already substantial global burden of micronutrient deficiencies. According to the World Health Organization (WHO), anaemia affected 30.7% of women aged 15–49 years in 2023 and 39.8% of children aged 6–59 months in the latest available global estimates (15). The burden is particularly pronounced in several regions, with nearly four in 10 women in the African and Eastern Mediterranean Regions affected by anaemia over the past decade (15). Furthermore, women of reproductive age in the WHO African and South-East Asia Regions collectively account for more than 60% of the global burden of anaemia in this population group. Similarly, vitamin A deficiency remains a major public health concern, with WHO estimates indicating that approximately 190 million preschool-aged children and 19.1 million pregnant women have subclinical vitamin A deficiency, with particularly high prevalence in Africa and South-East Asia (16). Zn-deficiency is more difficult to quantify because reliable population biomarkers are limited, but modelling has estimated inadequate Zn intake in about 17.3% of the global population, with the greatest risks concentrated in South Asia and sub-Saharan Africa (17). Together, these figures demonstrate the magnitude of existing micronutrient challenges and underscore the potential for climate change to further intensify nutritional vulnerabilities, particularly among populations already experiencing a high burden of deficiency.

Climate–nutrition relationship is more complex than a universal decline in food quality. Environmental stress can stimulate particular secondary metabolites, including phenolics, flavonoids, glucosinolates, carotenoids, and antioxidants, whereas severe stress may suppress biosynthetic processes (18, 19). Responses also vary among species, cultivars, developmental stages, and growing environments (18, 20). Thus, the central question is whether crops can maintain nutritionally meaningful outputs as climate conditions change. This mini-review addresses a gap in current climate–crop and nutrition discussions by bringing productivity, nutrient density, bioavailability, and dietary diversity into one crop-focused framework. The proposed concept of “nutritional resilience” does not replace established food-security or climate-resilience frameworks; rather, it specifies a nutrition-oriented outcome that can be assessed across the pathway from crop production to human consumption.

## 2 Climate change and crop nutritional quality

A central concern is that a crop may remain productive while becoming nutritionally less valuable. Elevated CO_2_ illustrates this dilemma clearly. In C3 crops, enhanced carbon assimilation can increase biomass and carbohydrate accumulation faster than mineral and protein acquisition, resulting in a nutrient-dilution effect (12, 13). Elevated CO_2_ can influence plant nutrient concentrations through several interacting physiological mechanisms. Increased CO_2_ generally reduces stomatal conductance and transpiration, which may decrease transpiration-driven mass flow of mobile nutrients such as nitrogen from the soil to plant roots. At the same time, enhanced carbon assimilation can increase carbohydrate accumulation and biomass production, potentially diluting nutrient concentrations when nutrient uptake does not increase proportionally with growth. Elevated CO_2_ may also alter root development, nutrient uptake capacity, and nutrient allocation within plants. Wheat, rice, and other C3 cereals have therefore been identified as particularly vulnerable to reductions in grain protein and micronutrient concentrations under elevated CO_2_ (10, 21). C3 cereals such as wheat, rice, and barley generally exhibit stronger CO_2_ fertilization responses than C4 crops. In contrast, maize, sorghum, millet, and sugarcane possess CO_2_-concentrating mechanisms and therefore generally show weaker direct growth responses to elevated CO_2_ (12, 22). Nevertheless, C4 crops can also experience nutritional disruption under combined heat, drought, and altered soil nutrient availability (21, 23). Importantly, the direction and magnitude of nutritional responses are not uniform. Effects can differ among crop species and genotypes, among nutrients, and according to stress intensity, duration, developmental stage, nutrient supply, and environmental context. For example, elevated CO_2_ may decrease protein and mineral concentrations in many C3 cereals, whereas selected secondary metabolites can increase under moderate heat, salinity, or other stresses (18–20). This distinction highlights an important conceptual shift: yield resilience and nutritional resilience are related but not synonymous traits. Climate adaptation strategies that select solely for biomass or grain yield may therefore overlook changes in protein and micronutrient density, as well as potentially beneficial or adverse changes in other nutritional components. Projections linking elevated CO_2_ to additional nutritional risk further illustrate the importance of this issue: atmospheric CO_2_ concentrations exceeding 500 ppm have been modelled to increase the number of people at risk of protein deficiency by 2050, although the magnitude of such projections depends on assumptions about dietary patterns, crop responses, and adaptation (24).

Heat stress affects nutritional quality through several interconnected pathways. Higher temperatures can accelerate respiration, shorten phenological stages, reduce grain-filling duration, and interfere with nutrient transport and assimilation (2, 21, 25, 26). In wheat, climate warming has been associated with reductions in grain protein and micronutrients, including Fe and Zn (21, 27). Drought imposes nutritional constraints through reduced soil moisture, lower nutrient mobility, impaired root growth, and restricted nutrient uptake. Water stress can also reduce biological nitrogen fixation in legumes, thereby affecting protein accumulation (2, 24, 25). Conversely, excessive rainfall and flooding can induce soil hypoxia, alter redox conditions, promote denitrification, and increase nutrient losses (2, 26). Importantly, combined stresses may be more consequential than individual stressors, yet multifactorial experiments remain comparatively scarce (18, 28, 29). Overall, climate effects on crop nutritional quality should be interpreted as conditional rather than uniformly detrimental, with outcomes determined by the interaction among crop genotype, nutrient, stressor, timing, intensity, duration, and environment. Climate-resilient nutrition therefore requires evaluation of nutrition stability of crops under realistic combinations of elevated CO_2_, temperature, water availability, salinity, and soil conditions.

## 3 Nutrient bioavailability and functional quality under climate stress

A critical extension of the climate–nutrition nexus is the distinction between nutrient concentration and nutritional value. The presence of a nutrient in a food does not necessarily indicate that it will be accessible during digestion or efficiently absorbed and utilized by the human body. Nutrient bioavailability is influenced by the plant matrix, interactions among nutrients, anti-nutritional compounds, food processing, and the physical accessibility of nutrients during digestion (18, 30, 31). Climate stress can alter these determinants as well as nutrient concentrations. Phytate, tannins and other polyphenols can complex minerals and reduce mineral bioavailability, while protease inhibitors and related proteinaceous anti-nutritional factors can affect protein digestion; oxalates can also bind minerals such as calcium (32, 33). Emerging evidence indicates that climatic responses of these compounds are highly context dependent. For example, elevated CO_2_ has decreased phytate in some wheat experiments but increased it in rice, whereas heat and drought have increased phytate in some wheat, rice, lentil, and other crops; genotype-specific responses have also been reported (33). Recent work therefore cautions against assuming that climate-induced changes in anti-nutritional factors will move in a single direction. Rather, changes in phytate, tannins, phenolics, oxalates, and protease inhibitors should be evaluated together with Fe, Zn, protein, amino acids, and measures of digestibility. Nutritional quality also extends well beyond protein and essential mineral nutrients such as Fe and Zn. Climate stress can substantially modify phenylpropanoid metabolism and the accumulation of phenolics, flavonoids, carotenoids, glucosinolates, vitamins, and other secondary metabolites, many of which contribute to antioxidant capacity, plant defence, sensory characteristics, and potential health-promoting properties (5, 6, 34). However, these responses are highly dependent on the type, intensity, and duration of stress, as well as crop species and genotype. For example, elevated temperature can alter the accumulation of phenolic compounds and flavonoids, whereas enhanced UV radiation may stimulate the synthesis of anthocyanins, flavonoids, vitamin C, and other protective metabolites in certain crops (5, 18, 35, 36). Similarly, moderate salinity stress can increase the accumulation of selected phenolics, glucosinolates, carotenoids, and antioxidant-related compounds in some vegetables, while severe or prolonged salinity could impair growth and nutritional quality (18, 37).

These contrasting responses highlight that climate stress does not necessarily result in a uniform deterioration of food quality; rather, it can reconfigure the nutritional, phytochemical, and functional profile of foods. An increase in the concentration of a bioactive compound, however, should not automatically be interpreted as an improvement in nutritional value, because its physiological significance ultimately depends on its stability during processing and digestion, bioavailability, metabolism, and biological activity in humans. Conversely, a reduction in nutrient concentration may be particularly consequential when it occurs alongside reduced bioavailability or when multiple nutrients and bioactive compounds are simultaneously affected. Consequently, climate-resilient food systems should adopt a more comprehensive definition of nutritional quality that integrates nutrient concentration, bioavailability, bioactivity, and functional properties. Future assessments should therefore evaluate nutritional outcomes across the entire pathway from soil to plant, plant to food, and food to human absorption and utilization, rather than relying exclusively on compositional measurements at harvest. Such a whole-food-system perspective is essential for determining whether climate-induced changes in crop composition ultimately translate into meaningful benefits or risks for human nutrition and health (Figure 1).

**Figure 1:**
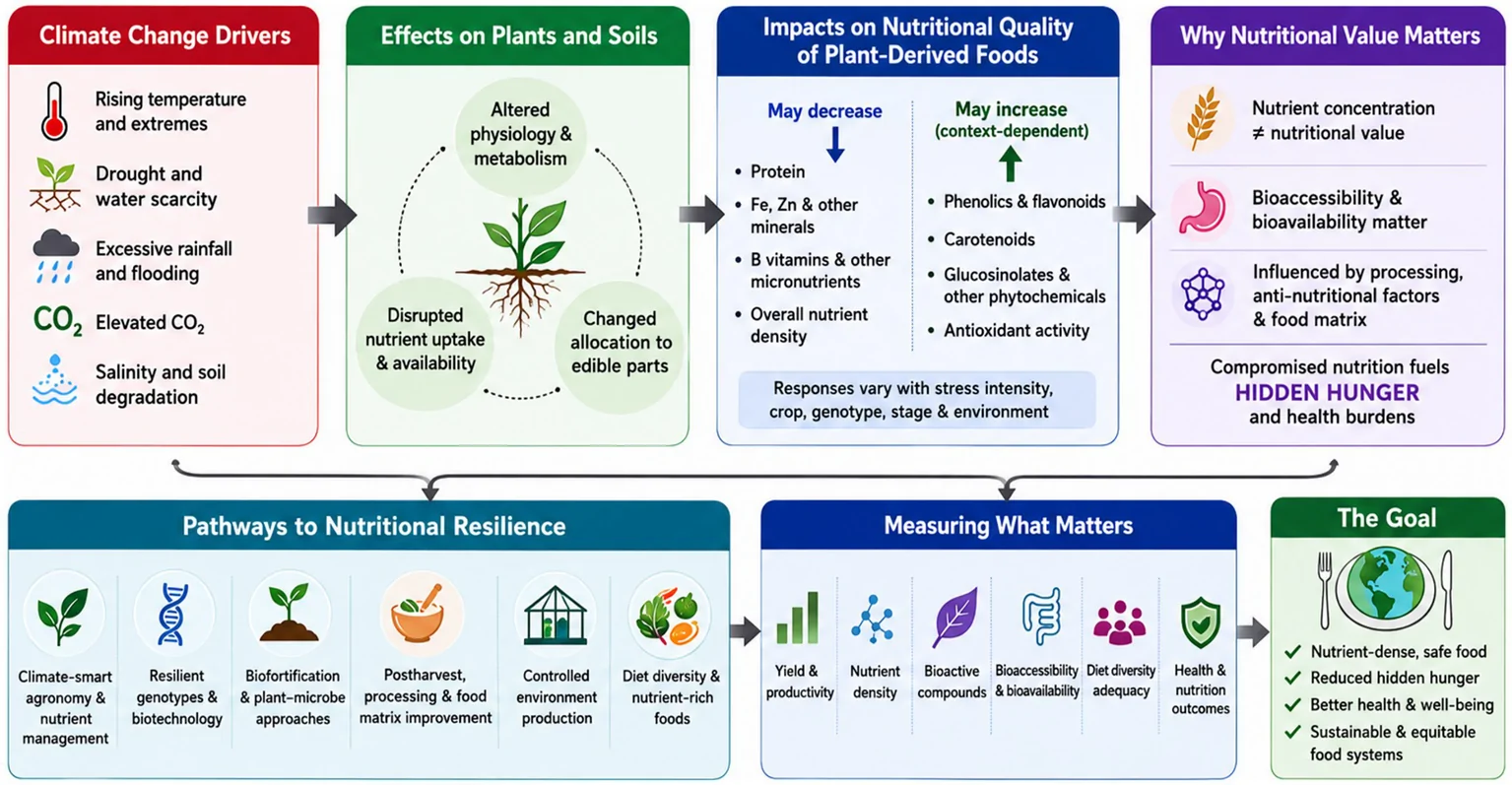
Conceptual framework for re-imagining crop nutritional resilience under climate change. The figure illustrates a shift from evaluating climate resilience primarily through yield stability toward evaluating nutritionally meaningful food delivery. The framework integrates four interconnected dimensions; (i) productivity, (ii) nutrient density, (iii) bioavailability, and (iv) dietary diversity, and links crop responses and management interventions with food quality and population-level nutritional adequacy. Dimensions (i) and (ii) collectively determine the total nutrient yield per unit land area, which can also be considered as a potential metric, if required. This figure was created with the assistance of ChatGPT through an iterative, prompt-based process to develop and refine the visual representation. The scientific concepts, interpretation, and overall message presented in the figure were developed and validated by the author.

## 4 Genotype × environment interactions for nutritional resilience

Variation among genotypes represents a valuable and largely underutilized opportunity for developing crop varieties capable of maintaining nutrient density under increasingly variable and stressful climatic conditions. Genotypes and cultivars can differ substantially in their physiological and nutritional responses to elevated CO_2_, warming, drought, and other environmental stresses, reflecting differences in their ability to sustain root growth, nutrient uptake and transport, nutrient transporter activity, and carbon allocation to developing tissues (12, 13). Such inherent variation could be exploited to identify and incorporate traits that minimize the decline in nutrient concentrations commonly associated with climate stress. For instance, some wheat cultivars maintain greater root biomass and higher nutrient transporter activity under elevated CO_2_, thereby enhancing nutrient acquisition and partially buffering the nutrient dilution effect associated with increased carbohydrate accumulation (13).

Naturally resilient crops and their wild relatives also represent important reservoirs of adaptive traits that could broaden the genetic basis of climate-resilient crop improvement. Crops such as sorghum, millet, and teff possess a range of physiological and morphological adaptations to arid and water-limited environments, while salt-tolerant plants offer additional genetic resources for improving crop performance under increasing soil salinity and other climate-related stresses (18, 38, 39). Importantly, these adaptive traits should be evaluated not only in terms of biomass production and yield stability but also for their potential to sustain the accumulation, transport, and storage of essential nutrients under stress. Conventional breeding, supported by modern genomic approaches and advanced biotechnology, including gene editing, provides opportunities to combine stress resilience with enhanced nutritional quality and nutrient-use efficiency (18, 40, 41). Future breeding programs should therefore adopt a more integrated selection strategy in which yield stability, stress tolerance, nutrient-use efficiency, and nutritional quality are considered simultaneously.

## 5 Biofortification for climate-resilient nutrition

Climate-resilient agriculture cannot be considered successful if it maintains crop yields while progressively reducing the nutritional value of food. This is particularly important because climate stress, declining soil fertility, nutrient dilution and limited dietary diversity can converge to intensify “hidden hunger” in populations dependent on staple crops (13, 42). Biofortification therefore provides a critical pathway for shifting the performance criterion of crops from “how much food is produced” to “how much nutrition is delivered per unit of food.” By increasing the concentration, uptake and/or bioavailability of essential nutrients during crop production, biofortification can embed nutritional security within agricultural systems rather than treating it as a post-harvest or health-sector intervention (43, 44). Three complementary strategies, breeding, agronomic management and biotechnology, provide different entry points for achieving this transition. Genetic biofortification through conventional and molecular breeding exploits natural variation to develop cultivars with elevated Fe, Zn, provitamin A, protein quality and other nutritional traits. Successful examples include Fe- and Zn-enriched wheat and rice, quality protein maize enriched in lysine and tryptophan, and provitamin A-rich orange-fleshed sweet potato (45–47). The integration of marker-assisted selection, quantitative trait loci and genomic approaches is further accelerating the identification and deployment of nutrient-associated alleles while offering opportunities to combine nutritional quality with yield and stress tolerance (48, 49).

Agronomic biofortification provides a faster and more readily deployable complement, particularly where genetically improved varieties require lengthy development or where biotechnology faces regulatory constraints (50–52). Soil and foliar applications of Zn, Fe, selenium and iodine can increase nutrient concentrations in edible tissues, while integrated soil–foliar management, organic amendments and beneficial microorganisms can improve nutrient availability, uptake and translocation (53, 54). Microbial inoculants are particularly promising because they can simultaneously enhance nutrient solubilization, plant growth and soil biological functioning. However, their effectiveness remains dependent on genotype, environment and field conditions, highlighting the need for locally adapted crop–microbiome combinations (Figure 1). Biotechnological approaches provide an additional opportunity where conventional genetic variation is insufficient. Genetic engineering and CRISPR/Cas-based genome editing can directly modify pathways governing nutrient uptake, transport, storage and biosynthesis. Examples include provitamin A enhancement in Golden Rice and targeted modification of genes influencing Fe, Zn and anti-nutritional phytate concentrations (55–57). Importantly, future biofortification should move beyond simply increasing nutrient concentration toward improving nutrient bioavailability, reducing anti-nutritional factors and simultaneously enhancing resilience to drought, heat and other stresses.

The strongest opportunity therefore lies not in choosing one biofortification strategy over another, but in integrating them. Nutrient-dense genotypes, precision nutrient management and beneficial microbial associations can be co-designed for specific agroecological environments. Such convergence can transform biofortification from a crop-improvement intervention into a systems-level strategy that delivers yield, nutritional quality and climate resilience together. The scale of deployment is substantial, for example, the HarvestPlus 2023 and 2024 Annual Reports reported that more than 458 varieties of 13 biofortified crops were available to farmers in more than 40 countries and that biofortified food production reached more than 360 million people; more than 115 million of these were farmers who grew and consumed biofortified crops on their farms (58, 59). The representative examples include Zn-enriched wheat, rice, and maize, Fe-enriched beans, and pearl millet, vitamin A-enriched cassava, maize, and sweet potato (Table 1). Thus, biofortification is increasingly a practical and scalable component of nutrition-sensitive agriculture, although deployment should continue to be evaluated for nutritional efficacy, adoption, climate adaptation, and local dietary relevance. The next generation of climate-resilient agriculture should therefore breed and manage crops not merely to survive climate change, but to continue nourishing people under it (Figure 1).

**Table 1:** Representative crops biofortified with targeted nutrients and their nutritional benefit.

Crop	Target nutrient	Nutritional benefit (% of daily adult requirement supplied)	Source
Rice	Zn	Up to 40%	(61, 62)
Wheat	Zn	Up to 50%	
Maize	Zn	Up to 70%	
	Vitamin A	Up to 50%	
Cassava	Vitamin A	Up to 100%	
Sweet potato	Vitamin A	Up to 100%	
Pearl millet	Fe	Up to 80%	
Bean	Fe	Up to 80%	

## 6 Diversifying the future food basket

A resilient food system should also diversify the sources from which nutrients are obtained. Controlled-environment agriculture can provide consistent production of nutrient-rich vegetables while reducing exposure to drought and other environmental stresses. Greenhouses, vertical farms, and hydroponic systems can provide greater control over water and nutrient supply, although energy requirements, capital costs, and scalability constrain their role in staple-crop production (60–64). Diversification can also be supported by underutilized and climate-resilient crops, including sorghum, millet, and teff, which are adapted to harsh environments (7), as well as by emerging alternative nutrient sources such as algae and edible insects. Microalgae such as *Chlorella vulgaris*, for example, contain protein, essential amino acids, vitamins, minerals, omega-3 fatty acids, and bioactive compounds (65). Edible insects can likewise provide protein, fatty acids, minerals, vitamins, and dietary fibre, although their wider use is influenced by consumer acceptance, cultural preferences, food safety, and regulatory considerations (66–69). These emerging options involve distinct technological requirements, evidence bases, costs, and scalability and are therefore better considered as complementary diversification pathways rather than substitutes for crop-based food production. In this crop-focused review, their role is considered only in the broader context of dietary diversification and nutritional resilience, rather than as core components of the crop nutritional-resilience framework.

## 7 Discussion and conclusions

The evidence reviewed here supports a more specific reframing of climate-resilient crop production. Resilience should be judged not only by whether crops maintain yield under stress, but also by whether they continue to deliver nutritionally meaningful food. Importantly, the evidence does not support a simple linear relationship between climate stress and poorer nutritional quality. Elevated CO_2_ frequently lowers protein and mineral concentrations in major C3 crops, while heat, drought, salinity, and other stresses can produce crop-, genotype-, nutrient-, and stage-specific increases or decreases in secondary metabolites and anti-nutritional factors (10–17, 32, 33). These changes in secondary metabolites are relevant to the framework because they can influence the nutritional quality, health-promoting properties, and bioavailability of nutrients in harvested crops, thereby affecting the overall nutritional value of food beyond its yield and primary nutrient content. The central implication is therefore one of conditional vulnerability: a crop may be productive yet nutritionally compromised, but a stress response may also preserve or enhance selected functional compounds. A practical framework for nutritional resilience should consequently incorporate four interconnected dimensions: productivity, nutrient density, bioavailability, and dietary diversity. These dimensions are not parallel check-boxes. Productivity determines the quantity of food available; nutrient density determines the amount of nutrient delivered per unit of food; bioavailability determines the fraction that can be released, absorbed, and utilized; and dietary diversity provides population-level buffering when a particular crop or nutrient source becomes vulnerable. The framework therefore links crop-level performance to food-level quality and population-level nutritional adequacy.

This four-dimension framework adds a nutrition-specific outcome layer to existing climate-resilience and food-security approaches. Conventional climate-resilience frameworks commonly emphasize persistence, adaptation, productivity, resource efficiency, or system stability, whereas food-security frameworks already encompass availability, access, utilization, and stability (1, 2). Nutritional resilience connects these perspectives by asking a more operational question: when climate conditions change, can the food system maintain nutritionally adequate and bioavailable foods while preserving productive capacity and dietary options? The reviewed evidence both supports and complicates this concept. It supports the framework because climate-driven nutrient dilution and altered bioavailability can occur without proportional yield losses. It complicates the framework because nutritional responses are multidirectional and because crop composition alone cannot predict human nutritional outcomes. Thus, nutritional resilience should be treated as a measurable outcome rather than as an assumption that follows automatically from yield resilience.

Taken together, the available evidence suggests that safeguarding crop nutritional quality under climate change will require an integrated rather than single-intervention strategy. Climate-smart agronomy, improved soil and water management, agronomic biofortification, nutrient-dense breeding, biotechnology, microbial biostimulants, controlled-environment production, and dietary diversification can be considered complementary components of a broader nutrition-resilient food system (Figure 1). Importantly, their effectiveness should be assessed not solely by their capacity to sustain yield, but whether they maintain nutritional outcomes across environments and through the food chain. Research priorities include multifactorial experiments combining heat, drought, elevated CO_2_, salinity, and nutrient stress; genotype × environment studies that explicitly measure nutritional traits; improved characterization of anti-nutritional factors; and stronger links between crop composition, dietary exposure, and human nutritional outcomes. These priorities are particularly important because the evidence base remains uneven across crops, nutrients, stress combinations, and geographic regions.

Finally, climate adaptation strategies and policies should move beyond production-centred metrics and explicitly incorporate nutritional resilience as a measurable dimension of food-system resilience. Indicators such as crop micronutrient density, nutrient retention, bioavailability, dietary diversity, and nutritional adequacy could complement conventional measures of yield, productivity, and resource-use efficiency in climate-smart agriculture programs. This positioning does not imply that nutritional resilience is a wholly new food-security paradigm; rather, it provides a crop- and food-quality lens through which existing resilience strategies can be assessed and improved. The central premise emerging from the literature is therefore that climate resilience should be evaluated together with nutritional outcomes. A food system cannot be considered resilient if it sustains harvests while progressively diminishing the nutritional value or functional properties of food. Moving “beyond yield” consequently requires an integrated framework connecting plant responses, soil and water management, crop improvement, food processing, nutrition, and dietary diversity. Ultimately, the objective is not simply to produce more food under climatic stress, but to preserve meaningful nutrient delivery and health-promoting functionality of foods from field to diet. Embedding this principle within research, breeding targets, agricultural policy, and food-system planning could strengthen the nutritional dimension of climate resilience (Figure 1).
